# Bacteremia caused by multidrug-resistant bacteria among hospitalized malnourished children in Mwanza, Tanzania: a cross sectional study

**DOI:** 10.1186/s13104-017-2389-z

**Published:** 2017-01-25

**Authors:** Maimuna Ahmed, Mariam M. Mirambo, Martha F. Mushi, Adolfine Hokororo, Stephen E. Mshana

**Affiliations:** 1 Department of Paediatrics and Child Health, Weill Bugando School of Medicine, Catholic University of Health and Allied Sciences, Mwanza, Tanzania; 2 Department of Microbiology and Immunology, Weill Bugando School of Medicine, Catholic University of Health and Allied Sciences, Mwanza, Tanzania

## Abstract

**Background:**

Severe malnutrition has been known to increase susceptibility and severity of infections. Bacteremia in malnourished children has been found to increase morbidity and mortality especially if is due to multidrug resistant bacteria. Here, we report the prevalence of bacteremia among children under 5 years of age and the antibiotic susceptibility pattern of the isolates; the information that can be used by clinicians to guide on the empirical antibiotic treatment.

**Findings:**

A total of 402 malnourished children were investigated for bacteremia. The median age of enrolled children were 17 (IQR 12–31) months. Severe malnutrition was observed in 19.1% of malnourished underfives. The point prevalence of bacteremia among malnourished children was 56/402 (13.9%; 95% CI 10.3–17.3). The prevalence of bacteremia was significantly higher among severely malnourished children than in children with moderate/mild malnutrition (18.0 vs. 10.7%, P = 0.03). Mortality was significantly associated with bacteremia among severely malnourished children (OR 2.77, 95% CI 1.02–6.98, P = 0.02). *Pseudomonas* spp. 20/56 (35.7%) were the most frequent isolates while *Staphylococcus aureus* and *Streptococcus pneumoniae* were isolated in 8/56 (14.2%) and 5/56 (8.9%) respectively. Rates of resistance for gram negative bacteria were; ampicillin (100%), amoxicillin/clavulanic acid (85.7%), gentamicin (23.8%), ceftriaxone (23.8%), ceftazidime (23.8%) meropenem (4.7%) and ciprofloxacin (2.4%). methicillin resistant *S. aureus* strains were confirmed in 4/8 (50%) of *S. aureus* isolates and 60% of *S. pneumoniae* isolates were resistant to 1 µg oxacillin.

**Conclusion:**

Bacteremia due to multi drug resistant isolates is common among severely malnourished children under 5 years of age. There is a need to review empirical antibiotic treatment coupled with antibiotic stewardship to prevent mortality and morbidity of severely malnourished children under 5 years of age.

## Findings

 Despite the efforts to reduce mortality among children under 5 years of age in resource limited countries; approximately 11 million children under 5 years of age die each year as a result of infections and malnutrition [1]. In low and middle income countries malnutrition has been found to cause high morbidity and mortality among children under 5 years of age [2–4]. Malnutrition has been known to suppress different components of immune system resulting into increased vulnerability and severity to infections [5]. In addition, severe malnutrition impairs both innate and acquired immune systems [6]. In addition, symptoms and signs of infections in malnourished children are often unremarkable making prompt clinical diagnosis and early treatment difficult [7–9]. Bacteremia is common among malnourished children and is associated with high mortality [10]. However, there is limited data regarding the prevalence of bacteremia due to multidrug resistant organisms among malnourished children in most centres in developing countries. This report for the first time in Mwanza, Tanzania shows the prevalence of bacteremia among malnourished children under 5 years of age at Bugando Medical Centre, in addition the antibiotic susceptibility pattern of the isolates from these children are also reported. This information is very important in the implementation of antibiotic stewardship and appropriate antibiotic empirical treatment in order to reduce the morbidity and mortality of malnourished children under 5 years of age.

A total of 402 malnourished children under 5 years of age were enrolled in this study between September 2012 and January 2013 at the Bugando Medical Centre (BMC). BMC is a tertiary, consultant and teaching hospital serving a population of approximately 13 million from the Victoria Lake zone of Tanzania. All malnourished children from the lake zone are referred to BMC for treatment. Malnutrition was assessed based on World Health Organization (WHO) criteria [11]. All children with congenital malformations and neurological disorders were excluded.

Demographic data and other factors related to malnutrition were collected using a structured pre-tested data collection tool. Nutritional status was assessed using WHO guidelines in which measurements of weight for length or height were interpreted using Z-score for mild (−1SD), moderate (−2SD) and severe (−3SD) malnutrition [11]. As per the BMC protocol, children with severe malnutrition and those suspected to have severe infections were admitted and received prophylactic ampicillin and gentamicin. For children with severe malnutrition were given F-75 during stabilization phase and F-100 and plumpynuts during rehabilitation phase. Moderately malnourished children were given plumpynuts while those with mild malnutrition received dietary counseling.

Blood samples from each participant were collected and processed as previously described [12]. Drug susceptibility testing was performed on pure colonies using disk diffusion method according to the Clinical and Laboratory Standard Institute CLSI [13]. Antibiotic discs tested were amoxicillin/clavulanic acid (20/10 μg), ampicillin (10 μg), gentamicin (10 μg), ciprofloxacin (5 μg), ceftazidime (30 μg), ceftriaxone (30 μg), and meropenem (10 μg) (Oxoid, UK). For gram positive bacteria; erythromycin (15 μg), clindamycin (2 μg) and oxacillin (1 μg) were tested. Cefoxitin (30 μg) was used to detect methicillin resistant *Staphylococcus aureus* (MRSA) while disk approximation method was done to confirm the presence of extended spectrum beta lactamases in *Escherichia coli*, *Klebsiella pneumoniae* and *Proteus mirabilis* as previously described [14]. *E. coli* ATCC 25922 and *S. aureus* ATCC 25923 were used for quality control of all microbiological tests.

Data were entered into a computer using Microsoft Excel 2007 and analyzed using STATA version 11 (college station, Texas). Chi square test was done to establish statistical difference for the categorical variables followed by logistic regression analysis to determine the strength of association between variables. Statistical significance was set at *P*  <  0.05 with a confidence interval of 95%.

The median age of enrolled children were 17 (IQR 12–31) months. Out of 402 children enrolled, 147 (36.6%), 77 (19.1%) and 178 (44.3%) had mild, moderate and severe malnutrition respectively. All 402 children were referred from various health facilities within the Victoria Lake zone. A total of 150/178 (84.2%) children with severe malnutrition had history of admission for more than 72 h. Oral antibiotic use (Trimethoprim/sulfamethoxazole and amoxicillin) was reported in 218/402 (54%) children including all 178 children with severe malnutrition. The point prevalence of bacteremia among malnourished children was 56/402 (13.9%; 95% CI 10.3–17.3). The prevalence was significantly higher among severely malnourished children than in children with moderate/mild malnutrition (32/178; 18.0% vs. 24/224; 10.7%, P = 0.03). Severely malnourished children were 1.83 more likely to have bacteremia than children with moderate/mild malnutrition (OR 1.83, 95% CI 1.03–3.23). No death occurred among 224 children with moderate/mild malnutrition while 31 (17.4%) deaths occurred among 178 severely malnourished children. Of 32 children with bacteremia, 10 (31.2%) died compared to 21 (14.4%) deaths from 146 children without bacteremia (OR 2.77, 95% CI 1.02–6.98, P = 0.02).

Of the 56 non-repetitive isolates from malnourished children, *Pseudomonas* spp. 20 (35.7%) were mostly frequent isolated, followed by *Enterobacter* spp. 9 (16.1%), *S. aureus* 8 (14.8%), *E. coli* 5 (8.9%), *Streptococcus pneumoniae* 5 (8.9%), *Streptococcus pyogenes* 1 (1.7%) and other gram negative spp. (*Shigella* spp., *Salmonella typhi*, *P. mirabilis*, *Acinetobacter* spp.*, Serratia* spp., and *K. pneumoniae*) which accounted for 8 (14.8%) of the isolates Table 1. Two *Pseudomonas aeruginosa* isolates 2/10 (10%) were found to be resistant to meropenem though the mechanisms of resistance could not be established. There was no data on the quantity of antibiotics use in the wards however; all children with severe malnutrition were admitted and received ampicillin and gentamicin. Out of 8 *S. aureus* isolates, 4 (50%) were found to be methicillin resistant *S. aureus* (MRSA) while 3/5 (60%) of *S. pneumoniae* were found to be resistant to oxacillin (1 µg). Two *S. pneumoniae* strains which were resistant to oxacillin (1 µg) were also resistant to clindamycin and erythromycin. Due to limited resources minimum inhibitory concentration (MIC) test was not done to confirm if these *S. pneumoniae* isolates were real resistant.

**Table 1:** Rate of resistance of 42 g negative bacteria isolated from malnourished children

Antibiotics							
Organisms	AMP	AMC	CN	CIP	CRO	CAZ	MEM
*Pseudomonas* spp. (20)	20 (100%)	17 (85%)	3 (15%)	0 (0%)		5 (25%)	2 (10%)
*E. coli* (5)	5 (100%)	4 (80%)	3 (60%)	1 (20%)	1 (20%)	1 (20%)	0 (0%)
*Enterobacter* spp. (9)	9 (100%)	7 (77.7%)	1 (11.1%)	0 (0.0%)	1 (11.1%)	1 (11.1%)	0 (0%)
^a^Others (8)	8 (100%)	8 (100%)	3 (37.5%)	0 (0%)	3 (37.5%)	3 (37.5%)	0 (0.0%)
Total (42)	42 (100%)	36 (85.7%)	10 (23.8%)	1 (2.4%)	10 (23.8%)	10 (23.8%)	2 (4.8%)

*AMP* ampicillin, *CN* Gentamicin, *CIP* ciprofloxacin, *AMC* amoxicillin/clavulanic acid, *CRO* ceftriaxone, *CAZ* ceftazidime

^a^Other gram negative (*Proteus* spp., *Enterobacter* spp., *Citrobacter* spp., *Serratia* spp.,)

Regarding resistant to third generation cephalosporins, the highest rate (37.5%) was observed among other gram negative bacteria. The rates of resistance to third generation cephalosporins for *E. coli* and *Enterobacter* spp. were 20 and 11.1% respectively.

Five isolates resistant to ceftazidime were from children with severe malnutrition; in addition, all two isolates resistant to meropenem were from severely malnourished children.

Out of 218 children who received prior antibiotics, 40 (18.3%) had bacteremia compared to 16/184 (8.5%) of those who did not receive prior antibiotics, P = 0.005. It should be noted that 81.6% of those with history of prior antibiotic use were severely malnourished. Out of 31 gram negative bacteria from children with prior history of antibiotic use, 28 (90.3%) were resistant to amoxicillin/clavulanic acid compared to 8/12 (66.7%) isolates from children without prior history of antibiotic use, P = 0.081. All isolates resistant to third generation cephalosporins were from children who received prior antibiotics treatment. Regarding gram positive isolates more resistance was also observed from children who received prior antibiotics (*S. aureus* 4/5 vs. 0/3, *S. pneumoniae* 3/4 vs. 0/1).

Bacteremia poses a potential risk of death among severely malnourished children. The risk becomes more significant when the bacteremia is due to multidrug resistant bacteria [12, 15]. Despite the fact that the prevalence of bacteremia is lower in this study as compared to previous studies; the most important findings in this study is bacteremia that is caused by multidrug resistant bacteria among severely malnourished children with the predominance of enteric gram negative bacteria [10, 16]. As previously documented, bacteremia can be caused by both gram positive and gram negative bacteria [17], however in this study multidrug resistant gram negative bacteria predominated as previously observed by Christopher et al. [18] in general paediatric population. The current study has observed *Pseudomonas* spp. to be most frequent isolates while in the previous study by Christopher et al. [18], *E. coli* and *K. pneumoniae* were found to be common isolates in the general paediatric wards. Majority of the isolates in this study were resistant to ampicillin and gentamicin, these are the first line treatment in most centres in developing countries necessitating review of treatment guidelines in these countries. About 25% of the isolates were resistant to the third generation cephalosporins, these antibiotics are considered as the second line treatment in most centres in developing countries. High rates resistance of third generation cephalosporins have been observed in previous studies among neonates and children under 5 years of age in our setting and other settings in Tanzania [12, 15, 18, 19]. This calls for an urgent establishment of antibiotic stewardship in developing countries to preserve these antibiotics for future generations. Inappropriate use of these antibiotics might be responsible for the increase in antimicrobial resistance that is currently being observed in many centres in developing countries. This is supported by the fact that in the current study low ciprofloxacin resistance rate was observed, the antibiotic which is not commonly used in paediatric wards in our centre.

As previously reported in Tanzania [20, 21] majority of *S. pneumoniae* strains were resistant to 1 µg oxacillin which predicts penicillin resistant *S. pneumoniae*. Despite the availability of vaccines for *S. pneumoniae* resistant strains not covered in the vaccine can cause severe infections with increased morbidity and mortality. *Staphylococcus aureus* has been found to cause bacteremia among children under 5 years of age [10]. In this study *S. aureus* was the commonest gram positive bacteria detected with 50% of the strains being MRSA. Infections caused MRSA strains have been associated with increased morbidity and mortality [12, 15, 22]. In developing countries, the treatment of MRSA is of great challenge because MRSA treatment requires antibiotics which are not commonly available in many centres. There is a need to invest more in controlling the emergence and spread of these strains in developing countries as cost effective measures.

Similarly to previous reports [23–25] mortality was significantly higher among severely malnourished children with bacteremia than in those with mild/moderate malnutrition. This is attributed to the impaired innate and adaptive immunity to fight against different infections [26].

Genotyping the pathogens to determine the clonality of these strains would have provided more information on the transmission pathways, however this was not done due to limited resources. In addition, the MIC for penicillin against *S. pneumoniae* was not done to confirm penicillin resistant *S. pneumoniae*.

## Conclusion

Multidrug resistant gram negative organisms are commonly causing bacteremia among severely malnourished children under 5 years of age. Culture and sensitivity is of great importance to guide appropriate empirical antibiotic treatment and to provide data that can be used for antibiotic stewardship programmes. A prospective study to investigate factors associated with multidrug resistant pathogens in severely malnourished children is highly needed.

## Abbreviations

BMC: Bugando Medical Centre
CREC: CUHAS/BUGANDO research ethics and review committee
CLSI: Clinical Laboratory Standard Institute
IQR: interquartile range
MRSA: methicilin resistant *Staphylococcus aureus*
OR: odds ratio
SD: standard deviation
